# The Microbial Metabolite Butyrate Induces Expression of Th1-Associated Factors in CD4^+^ T Cells

**DOI:** 10.3389/fimmu.2017.01036

**Published:** 2017-08-28

**Authors:** Meike Kespohl, Niyati Vachharajani, Maik Luu, Hani Harb, Sabine Pautz, Svenja Wolff, Nina Sillner, Alesia Walker, Philippe Schmitt-Kopplin, Thomas Boettger, Harald Renz, Stefan Offermanns, Ulrich Steinhoff, Alexander Visekruna

**Affiliations:** ^1^Institute for Medical Microbiology and Hygiene, Philipps University of Marburg, Marburg, Germany; ^2^Institute of Laboratory Medicine and Pathobiochemistry, Molecular Diagnostics, Philipps University of Marburg, Marburg, Germany; ^3^Research Unit Analytical BioGeoChemistry, Department of Environmental Sciences, Helmholtz Zentrum München, Neuherberg, Germany; ^4^ZIEL – Institute for Food and Health, Technical University of Munich, Freising, Germany; ^5^Analytical Food Chemistry, Technical University of Munich, Freising, Germany; ^6^Department of Cardiac Development and Remodelling, Max Planck Institute for Heart and Lung Research, Bad Nauheim, Germany; ^7^Department of Pharmacology, Max Planck Institute for Heart and Lung Research, Bad Nauheim, Germany

**Keywords:** short-chain fatty acids, butyrate, regulatory T cells, interferon-gamma, inhibition of histone deacetylase activity

## Abstract

Short-chain fatty acids (SCFAs), which are generated by the bacterial fermentation of dietary fibers, promote expansion of regulatory T cells (Tregs). Potential therapeutic value of SCFAs has been recently highlighted in the experimental models of T cell-mediated autoimmunity and allergic inflammation. These studies suggest that physiological intestinal concentrations of SCFAs within the millimolar range are crucial for dampening inflammation-mediated processes. Here, we describe opposing effects of SCFAs on T cell-mediated immune responses. In accordance with published data, lower butyrate concentrations facilitated differentiation of Tregs *in vitro* and *in vivo* under steady-state conditions. In contrast, higher concentrations of butyrate induced expression of the transcription factor T-bet in all investigated T cell subsets resulting in IFN-γ-producing Tregs or conventional T cells. This effect was mediated by the inhibition of histone deacetylase activity and was independent of SCFA-receptors FFA2 and FFA3 as well as of Na^+^-coupled SCFA transporter Slc5a8. Importantly, while butyrate was not able to induce the generation of Tregs in the absence of TGF-β1, the expression of T-bet and IFN-γ was triggered upon stimulation of CD4^+^ T cells with this SCFA alone. Moreover, the treatment of germ-free mice with butyrate enhanced the expression of T-bet and IFN-γ during acute colitis. Our data reveal that, depending on its concentration and immunological milieu, butyrate may exert either beneficial or detrimental effects on the mucosal immune system.

## Introduction

By populating the gut, several commensal species seem to safeguard the health of the organism by inducing differentiation and proliferation of regulatory T cells (Tregs) (1, 2). Short-chain fatty acids (SCFAs), which are microbial metabolites produced by commensal bacteria after fermentation of dietary fibers, are capable of expanding intestinal Treg population (3–5). It has been recently proposed that regulation of colonic Tregs by SCFAs *via* engagement of free fatty acid receptor FFA2 (GPR43) is one of the mechanisms by which commensal bacteria help to maintain intestinal immune homeostasis (6, 7). Aside from direct interaction of SCFAs with FFA2 on Tregs, butyrate has been shown to initiate differentiation of Tregs either *via* involvement of high-affinity SCFA transporter Slc5a8 in dendritic cells (DCs) or through activation of cell surface receptor GPR109a on colonic macrophages and DCs (8, 9). In addition, two recent studies demonstrated that the ability of SCFAs to promote induction of Foxp3 expression strongly correlated with their histone deacetylase (HDAC) inhibitory activity (3, 4). Collectively, these novel findings suggest that SCFAs exhibit pleiotropic effects on immune system, including the induction of Treg differentiation. Therefore, SCFAs such as butyrate, propionate, or acetate have recently been considered as potential therapeutic tool to modulate inflammatory responses (10, 11). Interestingly, increased consumption of dietary fibers led not only to SCFA-mediated expansion of Tregs but also suppressed the onset of carcinogenesis in the colon and dampened allergic responses in the lung of mice (8, 12). A recent study describing the impact of SCFAs on autoimmune reactions in the central nervous system (CNS) suggests that bacterial metabolites have a therapeutic potential for disorders such as multiple sclerosis (MS) (13). In spite of growing evidence of the immunomodulatory capacity for SCFAs, several questions concerning dosage and potential toxic effects remain unanswered. Furthermore, butyrate did not ameliorate inflammation in dextran sodium sulfate (DSS)-induced colitis model, thus raising the question if beneficial effects of SCFAs might be overridden by potentially deleterious, SCFA-mediated activities (14, 15).

In this study, we analyzed the dose-dependent impact of the microbial metabolite butyrate on Tregs and conventional CD4^+^ T cells. Low butyrate concentrations (0.1–0.5 mM) facilitated differentiation of Foxp3^+^ Tregs in the presence of TGF-β1, while, at a concentration of 1 mM, butyrate induced expression of T-bet and IFN-γ in Tregs and conventional T cells. Furthermore, during the DSS-induced colitis, the treatment of germ-free (GF) mice with butyrate increased the colonic expression of the pro-inflammatory molecules T-bet and IFN-γ.

## Materials and Methods

### Animals

6- to 10-week-old wild-type (WT) C57BL/6 mice were obtained from Charles River Laboratories (Sulzfeld, Germany) and were maintained under specific pathogen-free (SPF) or GF conditions. GF mice were kept in adequate isolators (Metall + Plastic, Radolfzell-Stahringen, Germany). The bedding, water, and food were routinely autoclaved, and the sterility of GF mice was checked biweekly. All experimental procedures with GF animals were carried out in a laminar flow hood under sterile conditions. *Ffar2^−/−^Ffar3^−/−^, Slc5a8^−/−^*, and *Tbx21^−/−^* mice on C57BL/6 background were bred at the animal facility of the Biomedical Research Center, University of Marburg, Germany. *Ffar2^−/−^Ffar3^−/−^* mice were generated by Prof. Stefan Offermanns (Max Planck Institute for Heart and Lung Research, Bad Nauheim, Germany) (16). *Slc5a8^−/−^* mice were received from Dr. Thomas Boettger (Max Planck Institute for Heart and Lung Research, Bad Nauheim, Germany). All animal experiments were conducted according to the German animal protection law.

### Isolation of CD4^+^ T Lymphocytes and *In Vitro* T Cell Differentiation

CD4^+^ T cells were purified from spleen and LNs and differentiated into Tregs or Th1 and Th2 cells *in vitro* as described previously (17). In brief, CD4^+^ T cells were isolated using the kit for negative isolation and were primed with plate-bound anti-CD3 (5 µg/ml, clone 145–2C11) and soluble anti-CD28 (1 µg/ml, clone 37.51) mAbs. In addition, 50 U/ml rh IL-2 (Novartis, Nürnberg, Germany) were provided for Th0, Th1, Th2, and Th17 cell cultures, whereas Tregs received 0.5 µg/ml anti-CD28 and 100 U/ml rhIL-2. Th1 cells were differentiated using 10 ng/ml rmIL-12 (PeproTech, Hamburg, Germany) together with anti-IL-4 (10% culture supernatant of clone 11B11). Th2 cells were generated by using 40 ng/ml rmIL-4 (PeproTech, Hamburg, Germany) together with 10 µg/ml anti-IFN-γ. For Th17 cultures, 0.5 ng/ml rhTGF-β1, 20 ng/ml IL-6 (both PeproTech, Hamburg, Germany), 10 µg/ml anti-IFN-γ, and anti-IL-4 (10% culture supernatant of clone 11B11) were added in the cell cultures. Optimal Treg conditions were obtained by addition of 10 µg/ml anti-IFN-γ, anti-IL-4 (10% culture supernatant of clone 11B11), and 2 ng/ml rhTGF-β1, whereas lower rhTGF-β1 concentrations (0.5 and 1 ng/ml) were used to achieve suboptimal Treg conditions. T cells were stimulated with 0.1–10 mM sodium butyrate, sodium propionate, sodium acetate, or 1–10 nM TSA (all substances from Sigma-Aldrich, München, Germany). Cells were cultured at 37°C for 72 h. Subsequently, the half of the cells was transferred to a new plate with 100 U/ml rhIL-2 plus the corresponding concentration of sodium butyrate, sodium propionate, sodium acetate, or TSA and rested for additional 48–72 h.

### Experimental Colitis and SCFA Administration

Following oral treatment with 100 mM sodium butyrate for 3 weeks, the acute colonic inflammation in GF mice was induced by adding 1.5% (w/v) DSS (MP Biomedicals, Eschwege, Germany) to the drinking water for 5 days (for the experiments with WT mice, 2% DSS was used). Animals were orally treated with 100 mM butyrate throughout the duration of the experiment based on the published protocol (6). In some experiments, mice were orally treated with 100 mM sodium butyrate for 3 weeks without colitis induction. In all SCFA experiments, the drinking water of control mice was pH- and sodium-adjusted. Colitis was scored using a previously published inflammation scoring system (18). Lamina propria mononuclear cells were purified from mice at day 8 after induction of colitis and were analyzed by FACS analysis and RT-PCR.

### Flow Cytometry

*In vitro* polarized CD4^+^ T lymphocytes were restimulated for 4 h with PMA (50 ng/mL)/ionomycin (750 ng/mL) in the presence of brefeldin A (10 mg/mL, all three substances from Sigma-Aldrich, München, Germany). For surface staining, fluorochrome-conjugated antibodies were added in appropriate dilutions and incubated for 20 min at 4°C. For intracellular staining of cytokines, CD4^+^ T cells were fixed with 2% formaldehyde solution and stained in 0.3% saponin buffer with the following antibodies: anti-IL-17A (eBio17B7), anti-IL-4 (11B11), and anti-IFN-γ (XMG1.2). For intracellular staining of transcription factors Foxp3 (FJK-16s) and T-bet (eBio4B10), the cells were fixed, permeabilized, and stained using Foxp3 staining kit (eBioscience, Frankfurt, Germany). Flow cytometry analysis was carried out on FACSCalibur (BD Bioscience, Heidelberg, Germany). Data were analyzed with FlowJo analysis software (TreeStar, Ashland, OR, USA).

### Immunoblot Analysis

For the preparation of cell lysates, 5 × 10^6^ cells were harvested from Treg-inducing cell cultures and pelleted at 390 × *g* for 10 min at 4°C. Cell pellets were lysed with RIPA cell lysis buffer for 20 min on ice. Following the SDS-PAGE, protein samples were transferred to a PVDF membrane, and protein detection was performed in a chemiluminescence image station (MicroChemi, Biostep GmbH, Burkhardtsdorf, Germany). For the detection of histone acetylation in Tregs, anti-acetyl-Histone H3 and H4 Abs (Merck Millipore, Darmstadt, Germany) were used. As a loading control for total cell protein extracts, a monoclonal anti-mouse β-actin Ab (Sigma-Aldrich, Munich, Germany) was used.

### HDAC Inhibitor Activity Assay

CD4^+^ T cells were cultured under Treg-inducing conditions for 72 h. Subsequently, the cells were harvested in the lysis buffer and subjected to HDAC inhibition by adding 5 mM of SCFAs for 10 min at room temperature. Following initial inhibition of HDACs, the peptide substrate Ac-Arg-Gly-Lys-AMC (Bachem, Bubendorf, Switzerland) was added to the reaction tubes for next 30 min and finally the stop solution stopped the reaction mediated by HDAC enzymes. HDAC inhibition activity was determined by measuring the fluorescence intensity of free AMC at the spectrofluorometer FLUOstar Omega (BMG Labtech, Ortenberg, Germany).

### Chromatin Immunoprecipitation (ChIP)

Chromatin immunoprecipitation analysis was performed on CD4^+^ T cells which were cultured under optimal Treg-polarizing conditions for 3 days. A total of 1 × 10^6^ Tregs were fixed with 1% formaldehyde for 10 min at room temperature. Subsequently, genomic DNA was extracted from precipitates using specific antibody for pan-acetylated H3 (Merk Millipore, Darmstadt, Germany). Control experiments were carried out with respective isotype antibodies as previously described (19). Samples were probed for the promoter regions of *Ifnγ, Il17a, Tbx21*, and *Foxp3* by quantitative RT-PCR using the following primers: *Ifnγ*, forward CATACCCTTTCCTTGCTTTTC and reverse TTGTGGGATTCTCTGAAAGCA, *Il17a*, forward TGGTTCTGTGCTGACCTCAT and reverse GCTCTCCCTGGACTCATGTT, *Tbx21* forward AGGTGGCAGGTTGACTCT and reverse CTGCTCCTGGGCTTTCTC, and *Foxp3*, forward TTCCCATTCAGCTTCA and reverse TGTTTGTGAGTGGAGG.

### qRT-PCR

In a first step, the total RNA is isolated from cell lysates. Subsequently, the RNA was transcribed into cDNA using the RevertAid First Strand cDNA Synthesis Kit (Thermo Scientific) according to the manufacturer’s instructions. Quantitative real-time PCR for *Tbx21, Ifnγ, Gata3*, and *Rorγt* was performed using a StepOnePlus device (Applied Biosystems, Darmstadt, Germany) with the following primers: *Tbx21* forward CAACAACCCCTTTGCCAAAG, *Tbx21* reverse TCCCCCAAGCAGTTGACAGT, *Foxp3* forward TTCCTTCCCAGAGTTCTTCCA, *Foxp3* reverse CATTGAGTGTCCTCTGCCTCT, *Ifnγ* forward GCAACAGCAAGGCGAAAAAG, *Ifnγ* reverse TTCCTGAGGCTGGATTCGG, *Gata3* forward AAGGCAGGGAGTGTGTGAAC, *Gata3* reverse AGGATGTCCCTGCTCTCCTT, *Rorγt* forward TCCTGCCACCTTGAGTATAGTCC, and *Rorγt* reverse GGACTATACTCAAGGTGGCAGGA. Quantitative analysis was performed by normalizing the target gene expression to expression of housekeeping gene *Hypoxanthine-guanine phosphoribosyl transferase* (*Hprt1*). The following primers were used for detection of *Hprt1*: forward, CTGGTGAAAAGGACCTCTCG, reverse, TGAAGTACTCATTATAGTCAAGGGCA.

### SCFAs Analysis by Means of Ultra-High Performance Liquid Chromatography–Mass Spectrometry (UHPLC-MS)

Luminal content of colon (~20 mg) was weighted in sterile ceramic bead tubes (NucleoSpin^®^ Bead Tubes, Macherey-Nagel, Dueren, Germany) and extracted with 1 mL chilled methanol (−20°C; LC-MS CHROMASOLV^®^, FLUKA, Sigma Aldrich, St Louis, MO, USA), containing 10 parts per million (ppm) of butyric acid-4,4,4-d3 (98 atom% D, Sigma Aldrich, St Louis, MO, USA) as internal standard. Homogenization and extraction were performed with Precellys^®^ Evolution Homogenizer (Bertin Corp., Rockville, Maryland, USA; 4,500 rpm, 40×3 s, 2 s pause time). Then, samples were centrifuged for 10 min at 21,000 × *g* and 4°C. SCFA standards including acetic, propionic, and butyric acids were all purchased from Sigma Aldrich (St Louis, MO, USA) and prepared in methanol to a stock solution concentration of 1,000 ppm. A SCFA working dilution of 100 ppm was prepared together with 100 ppm of butyric acid-4,4,4-d3. Derivatization of SCFAs was performed by using AMP + Mass Spectrometry Kit (Cayman Chemical, Ann Arbor, MI, USA). Eight microliters of SCFA working dilution, 100 ppm of butyric acid-4,4,4-d3, and extracted colon samples were derivatized according to manufacture descriptions of AMP + Mass Spectrometry Kit. Afterward, 50 µL of all samples were diluted with 405 µL of milliQH_2_O (milliQH_2_O, Milli-Q Integral Water Purification System, Billerica, MA, USA) to a final concentration of 1 ppm. A five-point calibration curve of SCFAs (0.05, 0.1, 0.2, 0.3, 0.4 ppm, each containing 0.1 ppm butyric acid-4,4,4-d3) was prepared in milliQH_2_O. Analyses of derivatized SCFAs and samples were performed with UHPLC (Acquity UPLC, Waters, Milford, MA, USA) coupled to MS. A reversed-phase separation was applied using a C8 column (C8: 1.7 µm, 2.1 mm × 150 mm, Acquity™ UPLC BEH™, Waters, Milford, MA, USA). Elution of derivatized SCFAs was ensured by following solvent system of ammonium acetate (5 mM, Sigma Aldrich, St Louis, MO, USA) combined with acetic acid (0.1%, pH 4.2, Biosolve, Valkenswaard, Netherlands) in water (A) (milliQH_2_O) and acetonitrile (B) (LC-MS CHROMASOLV^®^, FLUKA, Sigma Aldrich, St Louis, MO, USA). The flow rate was set to 0.3 mL/min, injection volume to 5 µL, and column temperature to 30°C. After a pre-runtime of 2 min, a gradient profile was applied by starting at 1% B for 1 min, increasing to 25% B until 10 min, following an increase to 95% B within 7 min. The 95% B was hold for 2 min, returning to initial 1% B within 0.2 min and further holding 2 min, resulting in a total run time of 22 min. The MS analysis (maXis, Bruker, Daltonics, Germany) was performed in positive electrospray ionization mode with the following parameters: nebulizer gas pressure of 2.0 bar, dry gas flow of 10.0 L/min, dry heater of 200°C, capillary voltage of 4,500 V, and end plate offset of −500 V. Theoretical m/z values and corresponding retention times of the derivatized SCFAs were the following: acetic acid ([M]+: 227.118438, 4.1 min), propionic acid ([M]+: 241.134088, 4.9 min), butyric acid ([M]+: 255.149738, 6.2 min), and butyric acid-4,4,4-d3 ([M]+: 258.16802, 6.2 min). Additionally, pseudo multiple reaction monitoring was used for butyric acid and butyric acid-4,4,4-d3 (isolation width of 10 Da). Acetic and propionic acid were also isolated and fragmented (isolation width of 10 Da and collision energy of 40 eV) to monitor typical loss of AMP compound in fragmentation experiments (MS/MS fragment with theoretical mass signal value [M]+: 169.0886). Calibration of mass spectrometer was ensured by injecting ESI-L Low Concentration Tuning Mix 100 mL (Agilent, Santa Clara, CA, USA) before the analysis. Peak areas and concentrations of SCFAs in luminal content of colon were elaborated with QuantAnalysis (Bruker, Daltonics, Bremen, Germany).

### Statistical Analysis

To compare the mean between two groups, statistical analysis was performed using an unpaired Student’s *t*-test with Prism 5 software (GraphPad, La Jolla, CA, USA). *P*-Values of *P* < 0.05 were considered statistically significant. For the comparisons of the multiple groups, the one-way analysis of variance was used. The following *P*-values were used: ***P < 0.001, **P = 0.001–0.01, *P = 0.01–0.05. Where appropriate, the mean ± SEM is represented in graphs.

## Results

### Butyrate Facilitates TGF-β1-Dependent Generation of Foxp3^+^ Tregs

**Figure 1:**
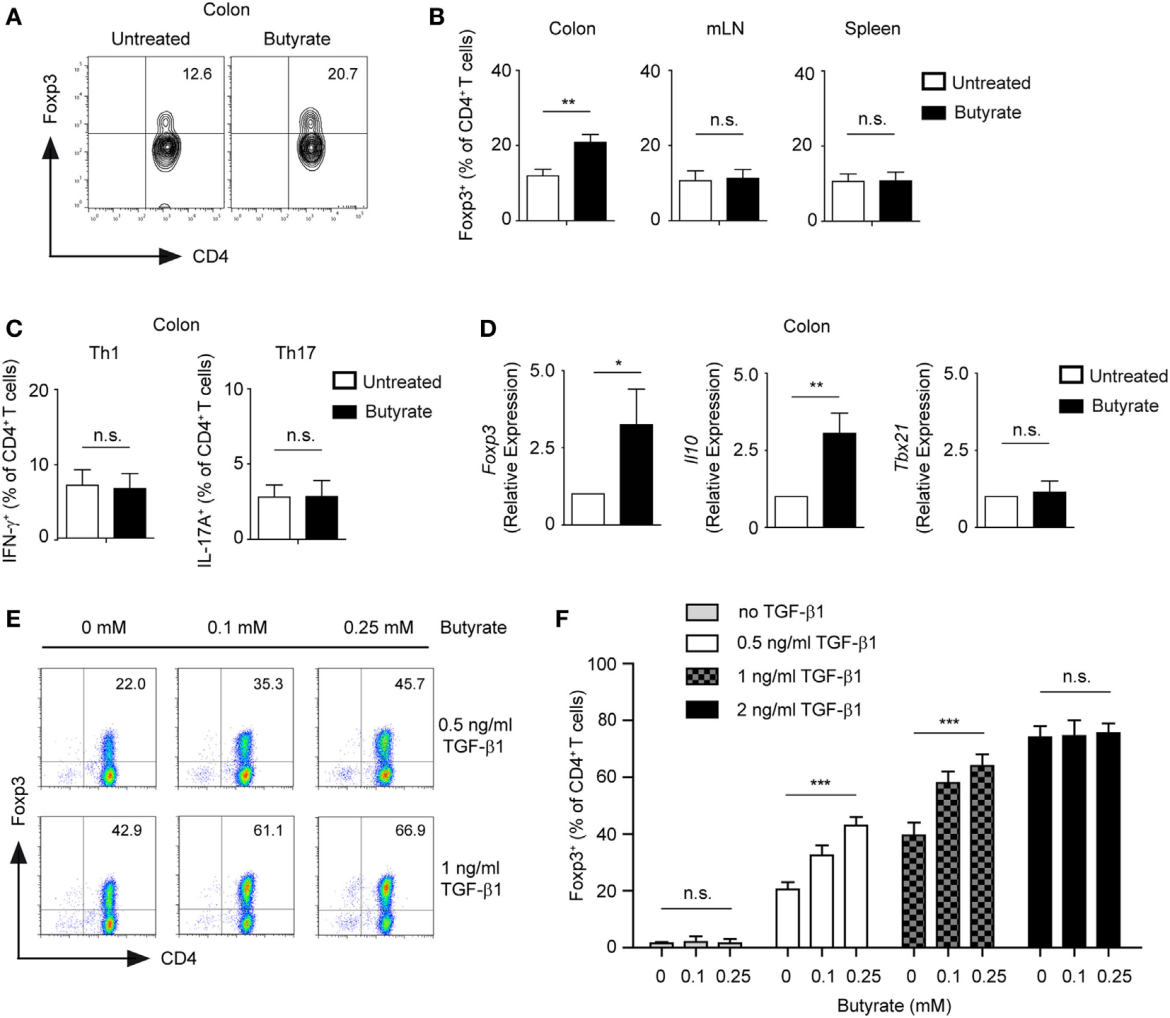
Butyrate promotes the expansion of Foxp3^+^ regulatory T cells (Tregs). **(A,B)** Wild-type (WT) mice were orally treated with 100 mM sodium butyrate for 3 weeks. The frequency of Foxp3^+^ Tregs was measured in colonic lamina propria, mesenteric lymph nodes, and spleen by FACS analysis. Bars represent mean ± SEM of two independent experiments; **P = 0.001–0.01, n.s., not significant. **(C)** The percentage of Th1 and Th17 cells within colonic CD4^+^ T lymphocytes was analyzed after 3 weeks of oral treatment of WT mice with 100 mM sodium butyrate. Data are shown as mean ± SEM; n.s., not significant. Two experiments were performed. **(D)** The relative expression of *Foxp3, Il10*, and *Tbx21* was analyzed by qRT-PCR in the colonic tissue of WT mice after oral treatment with butyrate (3 weeks, 100 mM sodium butyrate). Two similar experiments were performed. Data are the mean ± SEM; *P = 0.01–0.05, **P = 0.001–0.01, and n.s., not significant. **(E,F)** CD4^+^ T cells were cultured under Treg-inducing conditions in the presence of indicated concentrations of sodium butyrate and TGF-β1 for 5 days. Bars represent the mean ± SEM of three independent experiments; *** indicates *P* < 0.001, n.s., not significant.

Treatment of CD4^+^ T cells with butyrate has been shown to promote the differentiation of mucosal Tregs (3, 4). Accordingly, we observed that WT mice treated orally with 100 mM sodium butyrate for 3 weeks were capable of locally expanding Tregs in colonic lamina propria but not in mesenteric lymph nodes (mLN) or spleen (Figures 1A,B). A similar increase in the frequency of colonic Tregs was observed when mice received 200 mM of sodium butyrate in the drinking water. In contrast, no enhanced Treg responses were observed after oral treatment of WT animals with 50 mM sodium butyrate (Figure S1 in Supplementary Material). Furthermore, neither frequencies nor cell numbers of colonic Th1 and Th17 cells were affected by the butyrate treatment, suggesting that under steady-state conditions only colonic Tregs and not effector T cells are regulated by SCFAs (Figure 1C and data not shown). Finally, oral administration of butyrate resulted in increased colonic mRNA levels of *Il10* and *Foxp3* but not of pro-inflammatory genes such as *Tbx21* (Figure 1D).

In *in vitro* experiments, we confirmed that low concentrations of butyrate (0.1–0.25 mM) enhance expression of Foxp3 and differentiation of inducible (i) Tregs in the presence of TGF-β1 (Figure 1E). Interestingly, suboptimal Treg-inducing TGF-β1 concentrations were essential for butyrate-mediated impact on Treg differentiation. The treatment of CD4^+^ T cells with butyrate in the absence of TGF-β1 did not lead to conversion into Foxp3^+^ Tregs, whereas optimal Treg-inducing TGF-β1 concentrations masked the effects of butyrate (Figure 1F). To exclude potential contamination by low numbers of already pre-existing Tregs in the CD4^+^ T cell culture, we sorted and cultured highly purified naïve GFP^-^CD62L^+^CD4^+^ T cells from DEREG mice in the presence of butyrate and TGF-β1. After 5 days of culture, we observed an increase in GFP^+^CD4^+^ T lymphocytes in butyrate-treated cells as compared to the control cell cultures indicating a direct effect of butyrate on *de novo* generation of Tregs from naïve T cells (Figure S2 in Supplementary Material). Taken together, butyrate cooperates with TGF-β1 to enhance the expression of Foxp3 and differentiation into Tregs. This effect of butyrate was proposed to be based on its potent HDAC-inhibitory activity and ability to epigenetically regulate *Foxp3* gene expression. In addition, butyrate was also able to stabilize Foxp3 protein by promoting its acetylation (3, 20).

### Butyrate Induces Expression of T-bet and IFN-γ during Treg Differentiation

**Figure 2:**
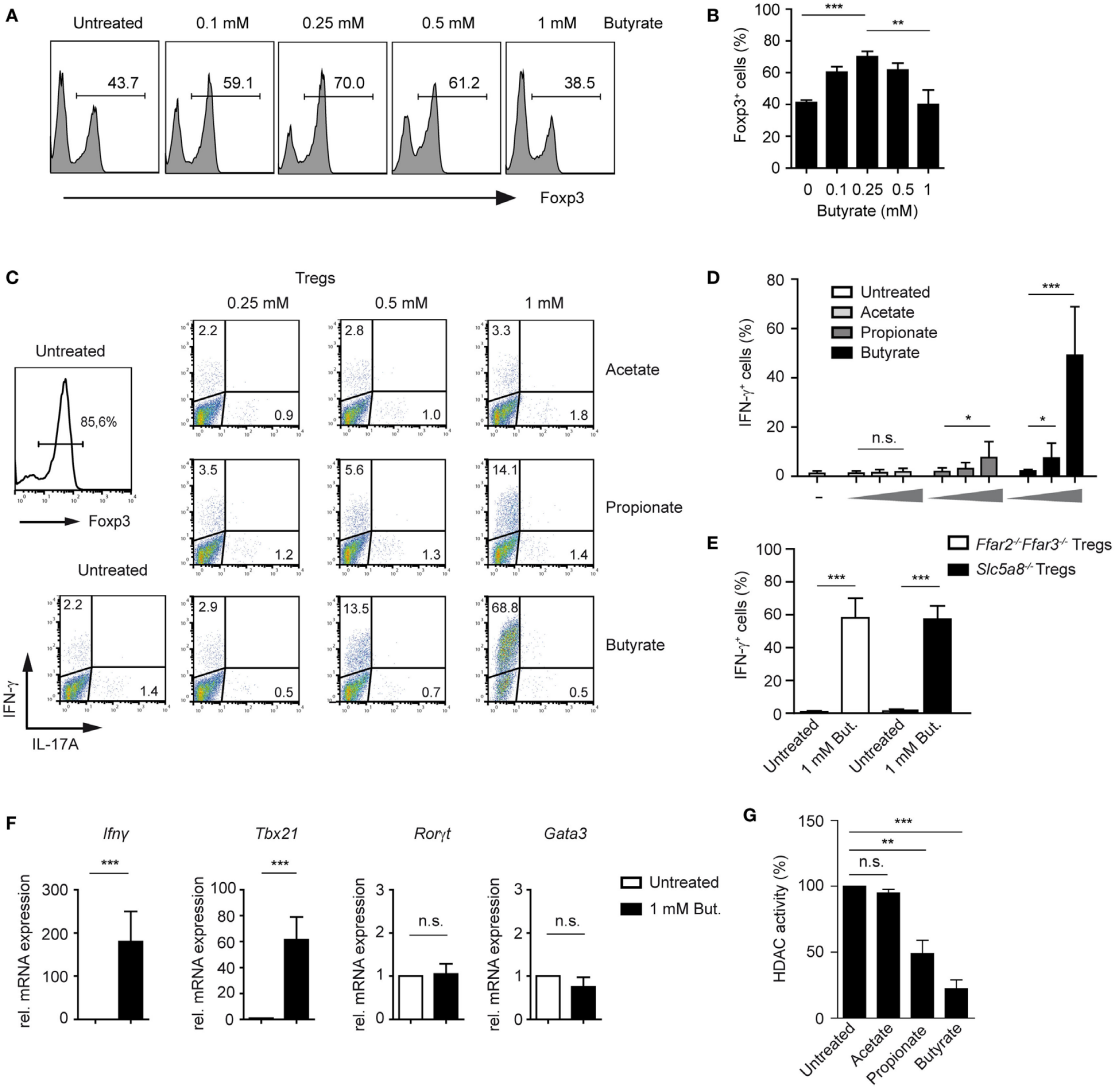
Butyrate induces the expression of IFN-γ and T-bet during the differentiation of regulatory T cells (Tregs). **(A,B)** The impact of increasing butyrate concentration on the Foxp3 expression was analyzed in T cells cultured under Treg-inducing conditions (1 ng/ml TGF-β1 and 100 U/ml rhIL-2) on day 6 of cell culture. The bars **(B)** represent mean ± SEM of three independent experiments; **P = 0.001–0.01, ***P < 0.001. **(C,D)** CD4^+^ T cells were differentiated into Tregs under optimal Treg-inducing conditions with 2 ng/ml TGF-β1 and 100 U/ml rhIL-2 in the presence of increasing concentrations of acetate, propionate, or butyrate. At day 6 of the cell culture, cells were restimulated for 4 h with PMA/ionomycin in the presence of Brefeldin A, stained for IFN-γ and IL-17A, and analyzed by flow cytometry. Bars **(D)** represent the mean ± SEM of IFN-γ^+^ cells. Two independent experiments were performed. *P = 0.01–0.05, ***P < 0.001, n.s., not significant. **(E)** CD4^+^ T cells isolated from LNs and spleens of *Ffar2^−/−^Ffar3^−/−^* and *Slc5a8^−/−^* mice were cultured under optimal Treg conditions for 6 days. The frequency of IFN-γ^+^ cells in the absence or presence of 1 mM sodium butyrate is shown. ***P < 0.001. **(F)** CD4^+^ T cells purified from WT mice were cultured under optimal Treg conditions for 3 days with or without 1 mM butyrate. The expression of *Ifnγ, Tbx21, Ror*γ*t*, and *Gata3* was analyzed by qRT-PCR. Data are the mean ± SEM of two independent experiments. ***P < 0.001, n.s., not significant. **(G)** CD4^+^ T cells were differentiated under optimal Treg conditions for 3 days and subsequently histone deacetylase (HDAC) inhibition assay in the presence of acetate, propionate, or butyrate was performed. Bars represent the mean ± SEM; **P = 0.001–0.01, ***P < 0.001, n.s., not significant.

Surprisingly, when performing a titration experiment with butyrate-treated T cells cultured under Treg-inducing conditions, we found that the frequencies of Foxp3 peaked at 0.25 mM butyrate concentration, while higher butyrate doses suppressed the differentiation of Foxp3^+^ Tregs (Figures 2A,B). Recently, it has been shown that SCFAs not only promote expansion of Tregs but impact also on Th1 and Th17 cells (21). Therefore, we examined whether SCFAs might trigger the production of pro-inflammatory cytokines under Treg-polarizing conditions. Notably, butyrate and to a lesser extent propionate were able to selectively induce expression of IFN-γ but not of IL-17A or IL-4 in a dose-dependent manner (Figures 2C,D; Figure S3A,B in Supplementary Material). Interestingly, this effect was independent of FFA2 and FFA3 receptors and of Slc5a8 transporter as the production of IFN-γ was not impaired in *Ffar2^−/−^Ffar3^−/−^* and *Slc5a8^−/−^* T cells during Treg differentiation upon butyrate treatment (Figure 2E). The percentage of Foxp3^+^ Tregs was similar in the spleen of WT, *Ffar2^−/−^Ffar3^−/−^*, and *Slc5a8^−/−^* mice and was also comparable in *in vitro* generated Treg cultures (Figure S4A–C in Supplementary Material). Both, *Ffar2^−/−^Ffar3^−/−^* and *Slc5a8^−/−^* CD4^+^ T cells cultured under Treg-inducing conditions and treated with butyrate behaved similar to butyrate-treated WT Tregs with respect to the Foxp3 expression (Figure S4D in Supplementary Material). Furthermore, quantitative RT-PCR data demonstrated that butyrate promoted induction of Th1-associated genes *Ifnγ* and *Tbx21* in T cells cultured under Treg-inducing conditions while the expression of Th17- and Th2-promoting transcription factors *Rorγt* and *Gata3* was not induced (Figure 2F). To test whether the increase in IFN-γ production correlates with the HDAC inhibitory effects of SCFAs, we performed an HDAC activity assay on iTregs in the presence of butyrate, propionate, and acetate. While butyrate and propionate exerted a potent HDAC inhibitory effect, acetate almost completely lacked this inhibitory activity (Figure 2G).

### Butyrate Induces H3 Acetylation at *Tbx21* and *Ifnγ* Locus during Treg Differentiation

**Figure 3:**
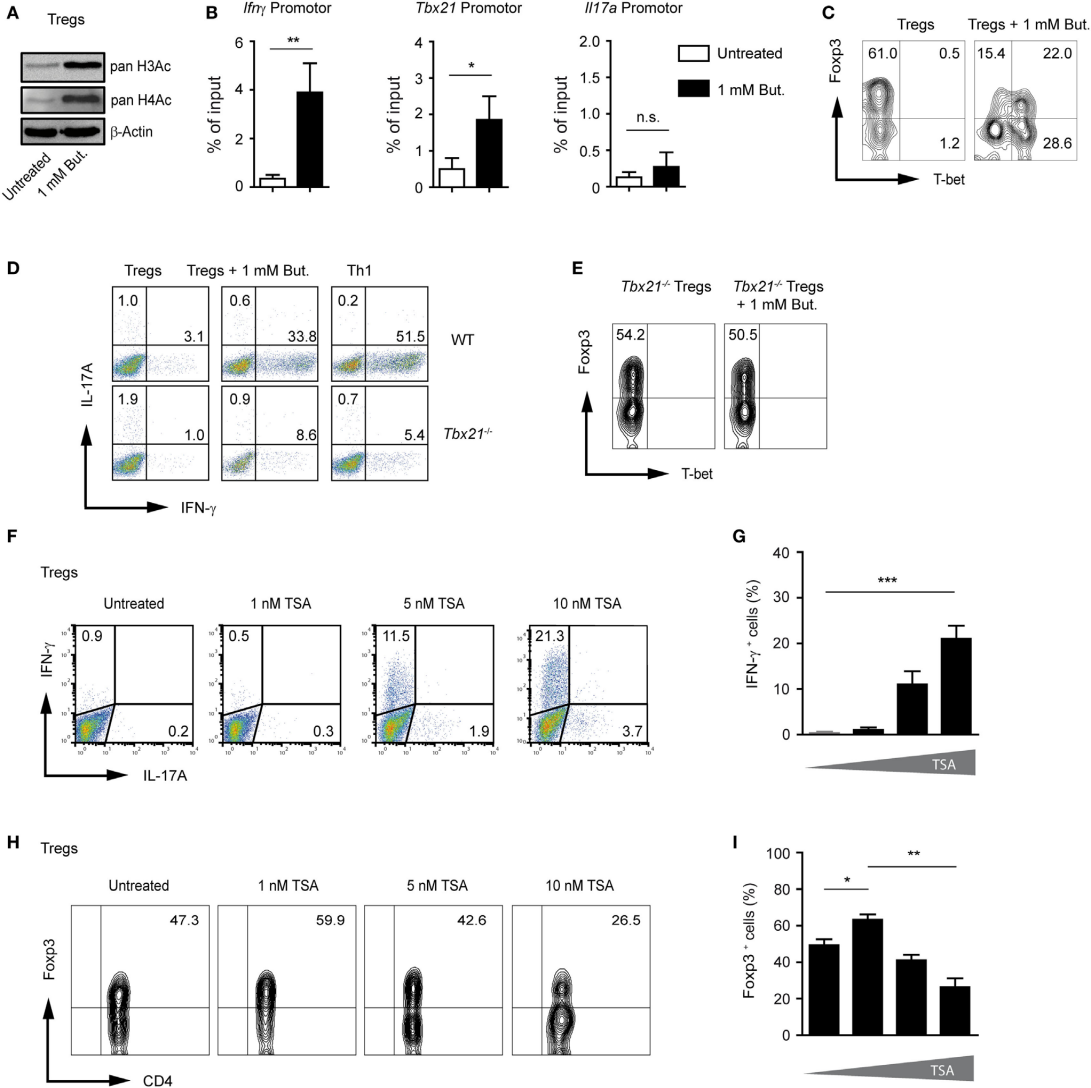
Butyrate induces changes in H3 acetylation at the *Ifng* and *Tbx21* locus during the differentiation of regulatory T cells (Tregs). **(A)** Western blot analysis showing the acetylation of histones H3 and H4 in the presence of 1 mM butyrate in CD4^+^ T cells cultured under Treg-inducing conditions for 3 days. Three similar experiments were performed. **(B)** Analysis of acetylated state of H3 at the promoter region of *Ifng, Tbx21*, and *Il17a* in T cells cultured under Treg-inducing conditions and treated with 1 mM butyrate for 3 days. Chromatin immunoprecipitation assay was performed using an anti-acetyl-H3 antibody. Data are average of two independent experiments; *P = 0.01–0.05, **P = 0.001–0.01, and n.s., not significant. **(C)** CD4^+^ T cells were differentiated into Tregs with 1 ng/ml TGF-β1 in the presence or absence of 1 mM butyrate. On day 3 of the cell culture, T-bet and Foxp3 expression was measured by FACS analysis. **(D)** Representative dot blots showing IFN-γ and IL-17A expression in wild-type (WT) and *Tbx21^−/−^* CD4^+^ T cells cultured under Treg- or Th1-inducing conditions for 3 days in the presence of 1 mM of sodium butyrate. One of three similar experiments is shown. **(E)** Representative dot blots showing T-bet and Foxp3 expression in *Tbx21^−/−^* CD4^+^ T cells cultured under Treg-inducing conditions with 1 ng/ml TGF-β1 in the presence 1 mM butyrate. **(F,G)** The frequency of IFN-γ^+^ and IL17A^+^ cells was determined on day 3 during the differentiation of Tregs in the presence of indicated TSA concentrations. Data **(G)** represent the mean ± SEM; ***P < 0.001. Two experiments were performed. **(H,I)** The percentage of Foxp3^+^CD4^+^ T cells was determined on day 3 during the differentiation of Tregs in the presence of indicated TSA concentrations. Results in **(I)** represent the mean ± SEM of two experiments; *P = 0.01–0.05, **P = 0.001–0.01.

As previously described, CD4^+^ T cells cultured under Treg-polarizing conditions and treated with 1 mM butyrate showed significantly increased acetylation of histones H3 and H4 as compared to untreated Tregs (Figure 3A). Under unpolarized conditions, histones are hypoacetylated at the *Ifnγ* locus in CD4^+^ T cells. During Th1 differentiation, proximal promoter sites of the *Ifnγ* gene become hyperacetylated (22, 23). To analyze if the inhibition of HDAC activity by butyrate induces histone acetylation directly at the *Ifnγ* and *Tbx21* locus during Treg differentiation, we performed ChIP assay using an anti-acetyl-H3 antibody. Our results revealed that butyrate was capable of promoting H3 acetylation at the *Ifng* and *Tbx21* but not at *Il17a* locus in T cells cultured under Treg-inducing conditions (Figure 3B). Of note, at a concentration of 0.25 mM, butyrate was already able to increase the pan-H3 acetylation during Treg differentiation (Figure S5A in Supplementary Material). In the absence of butyrate, at the *Foxp3* promoter region, H3 acetylation was observed only in TGF-β1-generated iTregs but not in naïve CD4^+^ T cells. This TGF-β1-mediated effect was enhanced after treatment of iTregs with 0.25 or 1 mM butyrate. Interestingly, at a concentration of 0.25 mM, butyrate was capable of upregulating H3 acetylation at the *Foxp3* but not at the *Ifnγ* and *Tbx21* promoter (Figure S5B,C in Supplementary Material).

T-bet and IFN-γ have been shown to directly oppose the induction of Foxp3 expression and negatively regulate differentiation of Tregs (24, 25). FACS analysis confirmed induction of T-bet protein and partial reduction of Foxp3 expression in butyrate-treated cells during Treg differentiation (Figure 3C). By comparing the ability of WT and *Tbx21^−/−^* CD4^+^ T cells cultured under Treg-inducing conditions to induce IFN-γ after butyrate treatment, we found that T-bet was required for production of endogenous IFN-γ (Figure 3D). Furthermore, we observed almost no reduction of Foxp3 frequencies in the absence of T-bet in T cells cultured under Treg-inducing conditions and treated with 1 mM butyrate. These data point to the role of this transcription factor in suppressing Foxp3 expression during the butyrate treatment (Figure 3E). To further prove the requirement of HDAC inhibition for IFN-γ induction, CD4^+^ T cells cultured under Treg-inducing conditions were treated with increasing concentrations of pan-HDAC inhibitor TSA. Indeed, the treatment with TSA caused the induction of IFN-γ production and reduction of Foxp3 expression during Treg differentiation (Figures 3F–I). In conclusion, our findings indicate that SCFAs might exhibit multiple effects during Treg differentiation including the induction of pro-inflammatory molecules.

### Butyrate Induces the Expression of IFN-γ in Unpolarized and Polarized CD4^+^ T Cells

**Figure 4:**
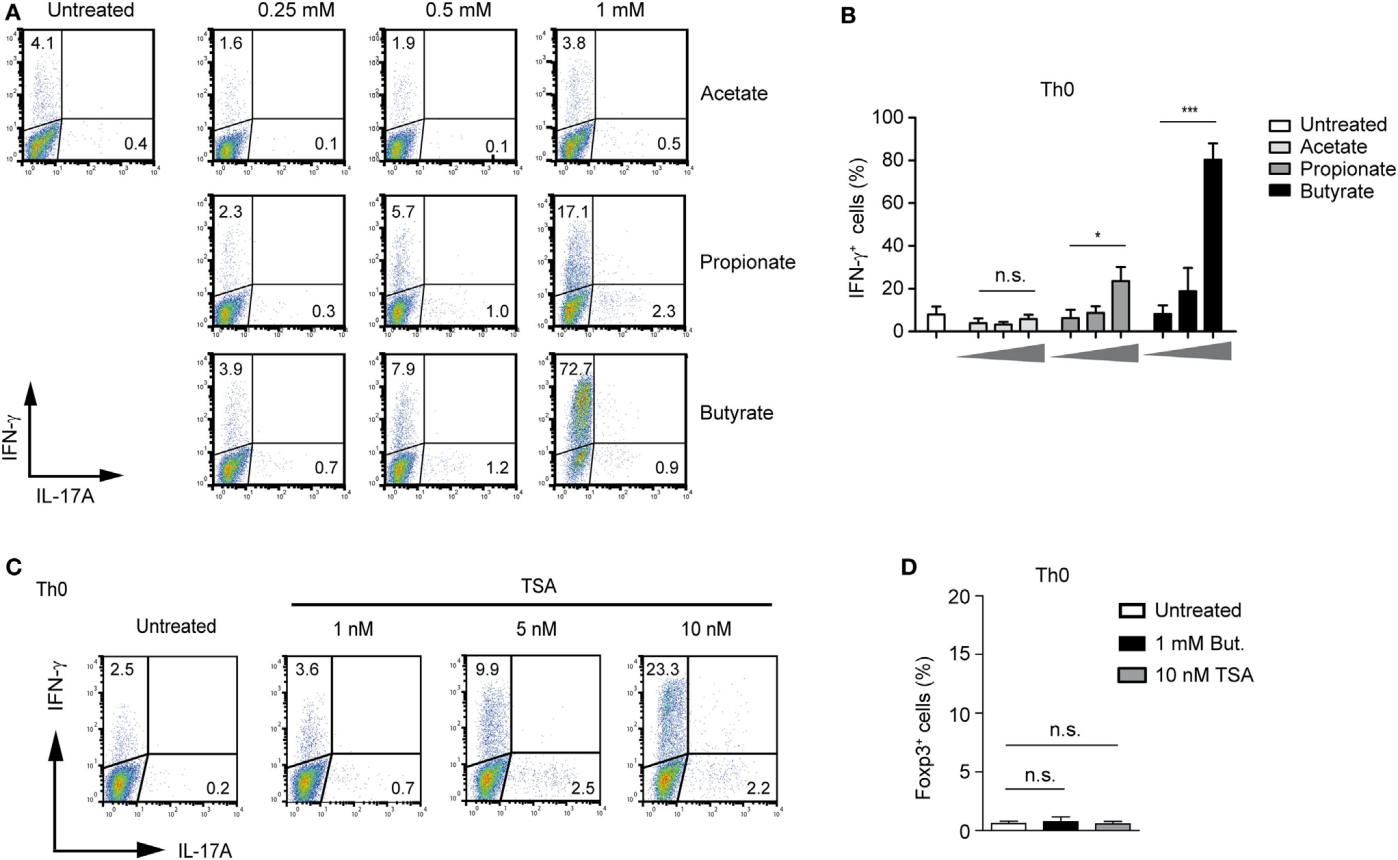
Butyrate induces IFN-γ expression in unpolarized CD4^+^ T cells. **(A,B)** FACS analysis of IFN-γ and IL-17A expression in CD4^+^ T cells cultured under unpolarized conditions in the presence of indicated short-chain fatty acid concentrations. Results in **(B)** represent the mean ± SEM; *P = 0.01–0.05, ***P < 0.001, n.s., not significant. Three independent experiments were performed. **(C)** CD4^+^ T cells were left unpolarized for 3 days in the presence of increasing TSA concentrations. Frequency of IFN-γ^+^ and IL-17A^+^ cells was determined using flow cytometry. Dot plots are representative of three similar experiments. **(D)** CD4^+^ T cells were left undifferentiated for 3 days in the presence of sodium butyrate and TSA, respectively. Intracellular staining for Foxp3 was performed by FACS analysis. Data are displayed as the mean ± SEM; n.s., not significant. Two experiments were performed.

To extend our analysis from the Treg-polarizing conditions to other effector T cells, we first examined possible effects of SCFA-induced IFN-γ expression under unpolarized conditions (Th0 cells). Butyrate was capable of selectively upregulating IFN-γ without inducing the expression of IL-17A (Figure 4A). The treatment of Th0 cells with butyrate increased the expression of IFN-γ in a dose-dependent manner peaking at the concentration of 1 mM. Propionate was less effective than butyrate, while acetate was not able to induce the expression of IFN-γ in unpolarized T cells (Figures 4A,B). Furthermore, we were wondering whether pan-HDAC inhibitor TSA is capable of inducing IFN-γ expression in Th0 cells. Similar to butyrate, a strong induction of IFN-γ but not of Foxp3 or IL-17A was observed after treatment of cells with TSA (Figures 4C,D). Of note, this effect of HDAC inhibitors butyrate and TSA was mediated without contribution of additional factors, while the butyrate-triggered expansion of Tregs was TGF-β1 dependent (Figure 1F).

**Figure 5:**
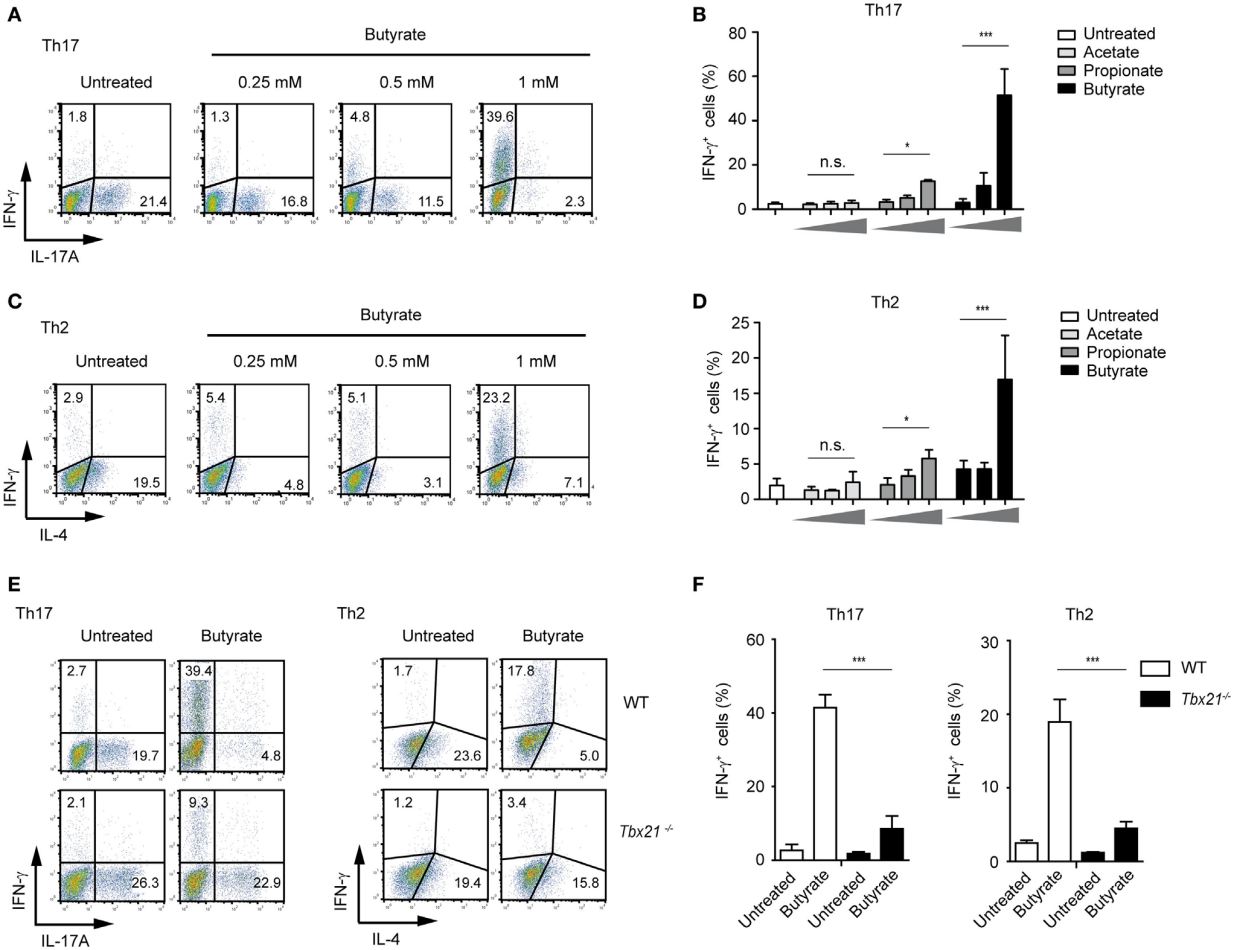
Impact of butyrate on Th17 and Th2 cells. **(A–D)** FACS analysis showing IFN-γ and IL-17A expression in CD4^+^ T cells cultured under Th17 conditions **(A,B)** or IFN-γ and IL-4 expression under Th2-polarizing conditions **(C,D)** for 6 days in the presence of indicated butyrate concentrations. Results in **(B,D)** represent the mean ± SEM of two experiments; *P = 0.01–0.05, ***P < 0.001, and n.s., not significant. **(E,F)** CD4^+^ T cells isolated from LNs and spleens of wild-type (WT) and *Tbx21^−/−^* mice were cultured under Th17- or Th2-polarizing conditions in the absence or presence of 1 mM butyrate. After 6 days of the cell culture, the FACS analysis for IFN-γ and IL-17A (Th17 cells) or IFN-γ and IL-4 (Th2 cells) was performed. Data **(F)** are displayed as the mean ± SEM obtained from two independent experiments; ***P < 0.001.

Moreover, in the presence of butyrate, the IFN-γ expression was increased even in Th1 cells that already display high levels of this cytokine (Figure S6 in Supplementary Material). Remarkably, we also observed a strong, butyrate-triggered induction of IFN-γ and T-bet and reduced IL-4 and IL-17A expression even under Th2- and Th17-promoting conditions, respectively (Figures 5A–D; Figure S7A in Supplementary Material). This effect seems to be mediated *via* T-bet as *Tbx21^−/−^* Th17 and Th2 cells did not switch toward IFN-γ production (Figures 5E,F). In accordance with these results, the inhibitory effect of butyrate on the expression of *Gata3* and *Rorγt*, genes encoding for transcription factors controlling Th2 and Th17 development, was detected in WT but not in T-bet-deficient cells (Figure S7B in Supplementary Material).

### Butyrate Increases T-bet and IFN-γ Expression Levels in Acute Colitis

**Figure 6:**
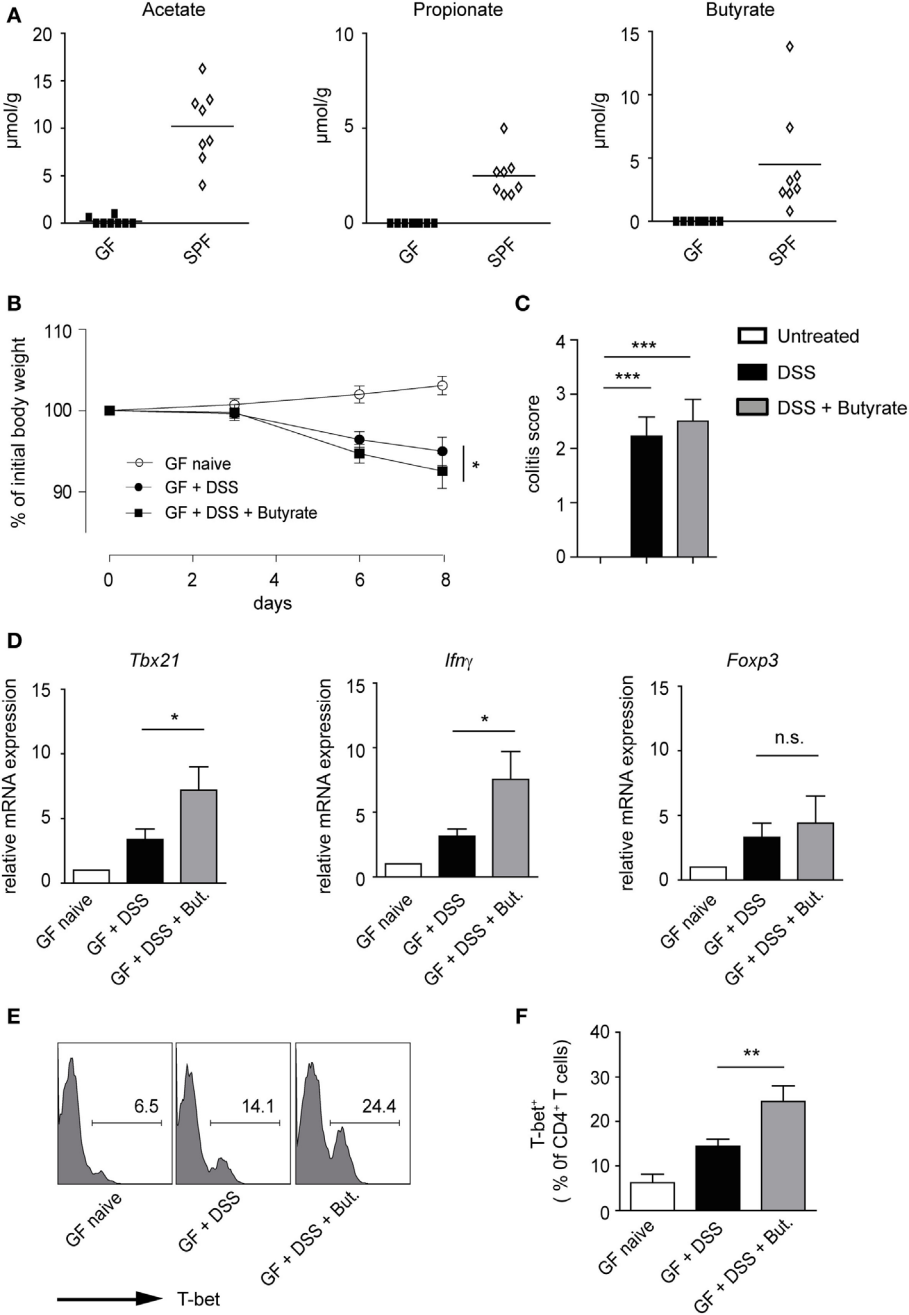
Impact of butyrate treatment on colonic acute inflammation in germ-free (GF) mice. **(A)** Short-chain fatty acid concentrations in colonic luminal content of GF and specific pathogen-free mice measured with ultra-high performance liquid chromatography–mass spectrometry. **(B,C)** GF mice were orally pretreated with 100 mM sodium butyrate for 3 weeks. Afterward, 1.5% DSS was given into drinking water for 5 days in the presence or absence of 100 mM butyrate. Changes in initial body weight **(B)** and determination of colitis score **(C)** on day 8 after DSS treatment are shown (*n* = 6 mice per group). Bars (C) represent the mean ± SEM; *P = 0.01–0.05, ***P < 0.001. **(D)** Acute colitis was induced in GF mice as described above. The colonic mRNA expression of *Tbx21, Ifnγ*, and *Foxp3* was analyzed on day 8 after colitis induction by qRT-PCR (*n* = 6 mice per group). Results are displayed as the mean ± SEM; *P = 0.01–0.05, n.s., not significant. **(E,F)** The percentages of T-bet^+^ cells within colonic CD4^+^ T lymphocytes on day 8 after induction of colitis in the indicated groups. Histograms **(E)** are representatives of two similar experiments showing the frequency of T-bet^+^ in CD4^+^ gate. Data **(F)** are presented as mean ± SEM, **P = 0.001–0.01.

A recent study has suggested that butyrate treatment is ineffective in ameliorating dextran sodium sulfate (DSS)-induced colitis in conventional mice pretreated with antibiotics (14). Although specific *in vivo* effects of SCFAs on Tregs have been proposed, the induction of Th1-associated genes by butyrate in an inflammatory milieu is also conceivable. To address this possibility, GF mice were orally treated with 100 mM butyrate [based on the published protocol (6)], during the course of DSS-induced colitis. As expected, in naïve GF mice, no SCFAs were found, owing to the absence of microbiota, whereas conventional (SPF) mice exhibited high luminal levels of acetate, propionate, and butyrate in the colon (Figure 6A). Our data demonstrate that butyrate treatment does not attenuate acute intestinal inflammation in GF animals. Although the severity of colitis was similar in both groups, GF mice treated with butyrate and DSS had even slightly increased weigh loss as compared to only DSS-administered mice (Figures 6B,C). While the treatment of animals with butyrate did not impact on the *Foxp3* levels during the course of inflammation, the expression of pro-inflammatory molecules *Tbx21* and *Ifnγ* was significantly increased after butyrate administration as compared to only DSS-treated group (Figure 6D). In addition, the FACS analysis confirmed a higher frequency of T-bet^+^ cells within colonic CD4^+^ T lymphocytes after butyrate treatment of GF mice (Figures 6E,F). A similar trend was observed in WT mice treated with butyrate and DSS (Figure S8A,B in Supplementary Material). In summary, our present data challenge the current paradigm that butyrate-mediated effects on the host immune system are only beneficial. We conclude that, in the context of inflammatory bowel disease (IBD), the elevated levels of butyrate in lamina propria might be more harmful than previously thought.

## Discussion

In the intestine, dietary fibers are fermented by commensal bacteria into SCFAs (26). Several beneficial effects of SCFAs such as suppression of intestinal inflammation, protection from colorectal cancer, and dampening allergic and autoimmune responses in the lung and CNS have been reported (6, 8, 13). Therefore, physiological concentrations of butyrate, propionate, and acetate (between 10 and 100 mM in the gut lumen) are thought to be essential for maintaining an immunological balance at mucosal surfaces. Recently, it has been shown that SCFAs promote expansion of colonic Tregs in conventionally reared and GF mice. Mechanistically, these effects of SCFAs were mediated through SCFA-receptor FFA2. In contrary to other Treg subpopulations, colonic Tregs were shown to express high levels of FFA2 and the observed SCFA-mediated effects were abrogated in mice lacking this receptor (6). Ability of butyrate to impact on differentiation of Tregs is likely mediated through its HDAC inhibitory activity because similar influence on Tregs was previously observed for pan-HDACi TSA (27, 28). In this study, we investigated the immunomodulatory effects of butyrate on Tregs and other CD4^+^ T cell subsets. Here, we demonstrate that butyrate is capable of triggering expression of T-bet and IFN-γ in all examined T cell populations. We further showed that GF mice treated with butyrate exhibited increased levels of pro-inflammatory molecules IFN-γ and T-bet during acute colonic inflammation. Thus, under homeostatic conditions, the coevolution of microbial community in the gut and mucosal immune system is reflected in the expansion of anti-inflammatory Tregs by microbial metabolites. Such anti-inflammatory activities of SCFAs and other metabolites might be overridden during the acute or chronic inflammation whereby SCFA-mediated pro-inflammatory effects might predominate.

In contrast to the previous work that has demonstrated that butyrate mainly modulates the histone acetylation at the *Foxp3* locus but not at the *Tbx21, Rorγt*, and *Gata3* genes (4), our novel data reveal that the promoters of some pro-inflammatory genes such as *Ifnγ* and *Tbx21* are hyperacetylated by butyrate under Treg-inducing conditions. These discrepant findings might be due to different doses of butyrate used in the experimental protocols. Previously, butyrate and pan-HDAC inhibitor TSA were shown to establish the long-range histone acetylation across the *Ifnγ* gene in CD4^+^ T cells under neutral conditions or were even able to restore the histone acetylation of the *Ifnγ* and *Tbx21* locus under Th2 conditions (23, 29). These findings might explain the underlying mechanisms involved *in vivo* observations made by us and others showing that SCFAs are able to exacerbate DSS-induced colitis or to cause T cell-mediated tissue inflammation in the renal system (15, 30).

Interestingly, physiological concentrations of SCFAs apart from the gut lumen and portal vein have not been assessed yet. Given that a large amount of luminal butyrate is consumed by colonic epithelial cells as energy source, physiological concentrations of butyrate in lamina propria are considered not to reach substantial levels. Alterations in intestinal permeability in patients with IBD due to disturbed epithelial barrier could lower the luminal levels of SCFAs and result in the influx of microbial metabolites in the lamina propria. In order to better understand various effects of SCFAs on the mucosal immune system, the measurement of the local concentrations of these metabolites in the intestinal-associated tissues such as mLN and Peyer’s patches at steady-state and under inflammatory conditions should be addressed in future studies. Collectively, we show that Foxp3^+^ Tregs require the presence of TGF-β1 for butyrate-mediated expansion. In contrast, the butyrate treatment of CD4^+^ T cells in the absence of additional stimuli is sufficient for rapid and robust induction of Th1-associated factors T-bet and IFN-γ.

## Ethics Statement

All animal experiments were conducted according to the German animal protection law. The study was approved by Regierungspräsidium Gießen, Germany, Nr. 70/2014.

## Author Contributions

MK, NV, ML, SP, and SW performed *ex vivo* and *in vitro* analyses. The *in vivo* experiments were done by ML, NV, and HH. HH performed the ChIP experiments. The measurement of SCFA concentrations in intestinal lumen was performed by NS, AW, and PS-K, TB, HR, and SO provided us with reagents and *Ffar2^−/−^Ffar3^−/−^* and *Slc5a8^−/−^* mice, respectively. AV and US conducted the study and AV wrote the manuscript. US, TB, HR, and SO critically reviewed the manuscript.

## Conflict of Interest Statement

The authors declare that the research was conducted in the absence of any commercial or financial relationships that could be construed as a potential conflict of interest.
