# Supplementary material for: The Microbial Metabolite Butyrate Induces Expression of Th1-Associated Factors in CD4+ T Cells
Source: Front Immunol. 2017 Aug 28;8:1036. doi: 10.3389/fimmu.2017.01036 (PMC5581317; doi:10.3389/fimmu.2017.01036)
Supplement: Supplementary file 1 [file Data_Sheet_1.PDF]

Supplementary Figures:

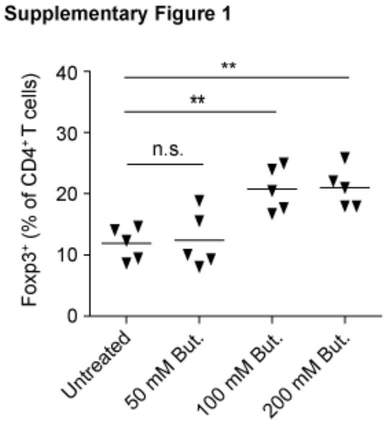

Supplementary Figure 1. WT mice orally treated with indicated butyrate concentrations for 21 days. The frequency of $Foxp3^{+}CD4^{+}$ cells was determined by flow cytometry analysis. For the statistical analysis, the one-way analysis of variance (ANOVA) was used. Bars represent the mean ± SEM; n.s. = not significant. **, P = 0.001-0.01.

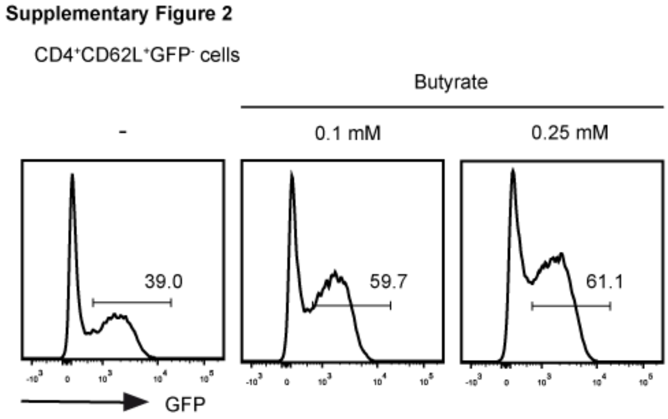

Supplementary Figure 2. Naïve $CD4^{+}CD62L^{+}GFP^{-}$ T cells were sorted from LN and spleen of DEREG mice and differentiated into Tregs in the presence of indicated concentrations of sodium butyrate. The frequencies of GFP^{+} ($Foxp3^{+}$) cells was determined by FACS analysis on day 5 of cell culture. Two similar experiment were performed.

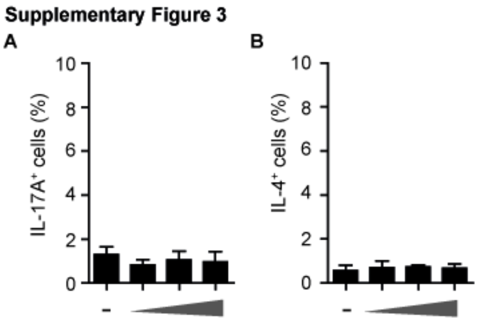

Supplementary Figure 3. (**A** and **B**) CD4$^+$ T cells were differentiated into Tregs in the presence of increasing concentrations of sodium butyrate (0, 0.25 mM, 0.5 mM and 1 mM). At day 6 of the cell culture, the intracellular staining for IL-4 and IL-17A was performed. Bars represent the mean ± SEM of IL-17A$^+$ (**A**) and IL-4$^+$ (**B**) T cells, respectively. Three independent experiments were performed.

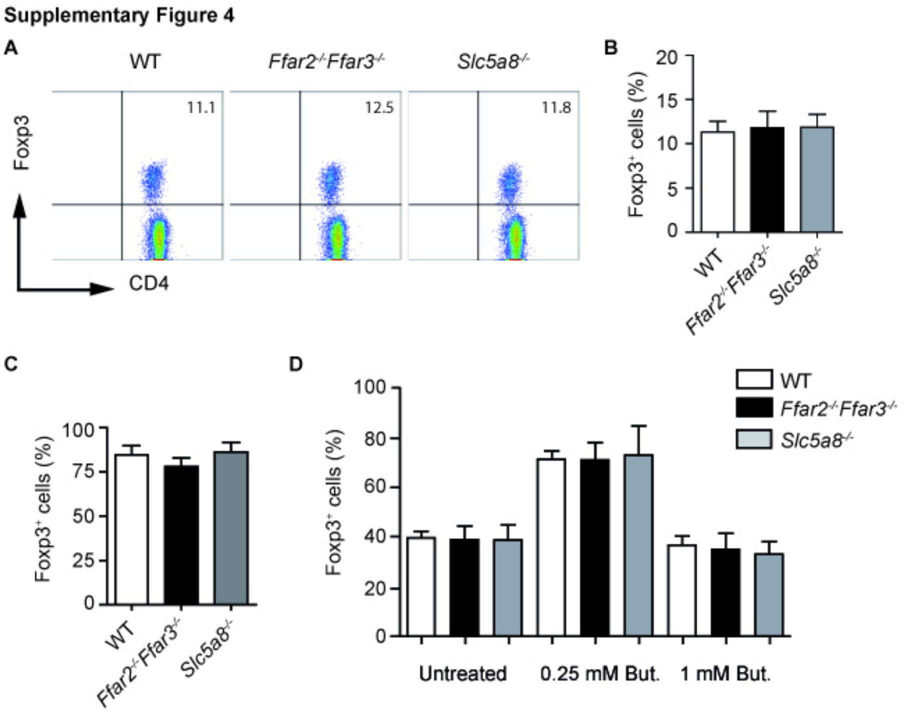

Supplementary Figure 4. (**A** and **B**) The percentage of Foxp3$^+$ Tregs within splenic CD4$^+$ T cell population in WT, *Ffar2*$^{-/-}$*Ffar3*$^{-/-}$ and *Slc5a8*$^{-/-}$ mice was analysed by FACS analysis. Two similar experiments were performed. (**C**) CD4$^+$ T cells isolated from LNs and spleens of WT, *Ffar2*$^{-/-}$*Ffar3*$^{-/-}$ and *Slc5a8*$^{-/-}$ mice were cultured under optimal Treg conditions (2 ng/ml TGF-β1 and 100 U/ml rhIL-2) for three days. The frequency of Foxp3$^+$ cells was analysed by flow cytometry. Bars represent the mean ± SEM. (**D**) WT, *Ffar2*$^{-/-}$*Ffar3*$^{-/-}$ and *Slc5a8*$^{-/-}$ CD4$^+$ T cells were cultured under Treg-inducing conditions (1 ng/ml TGF-β1 and 100 U/ml rhIL-2) and the percentage of Foxp3$^+$ cells was determined by FACS analysis. Bars represent the mean ± SEM. Two experiments were performed.

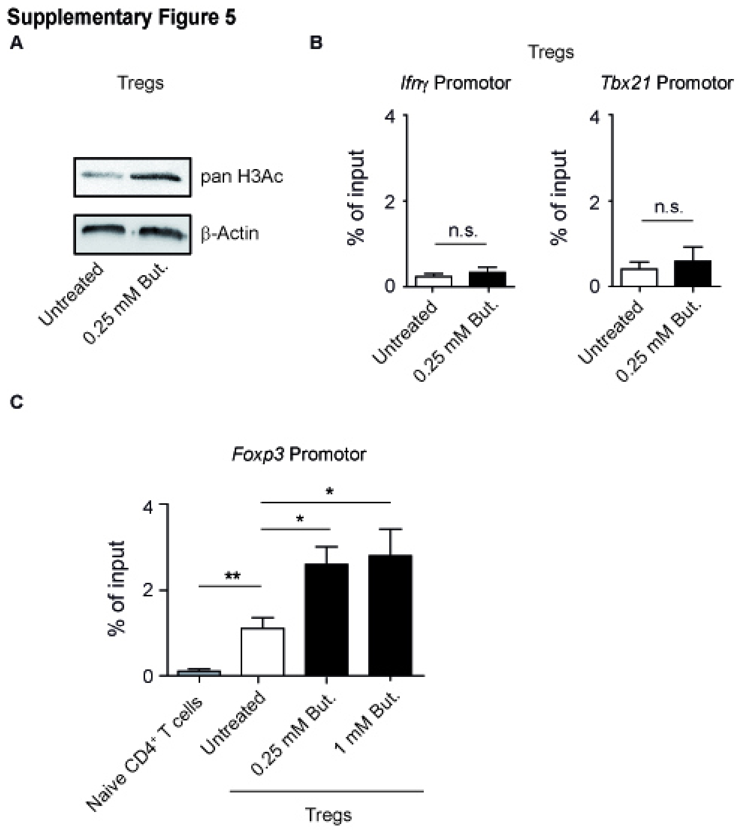

Supplementary Figure 5. (**A**) CD4$^+$ T cells were isolated from WT mice and cultured under Treg-inducing conditions in the presence or absence of 0.25 mM butyrate for 3 days. Western blot analysis shows the pan-acetylation of histones H3. Two experiments were performed. (**B** and **C**) ChIP analysis of acetylated state of H3 at the promotor region of *Ifnγ*, *Tbx21* and *Foxp3* in CD4$^+$ T cells cultured under Treg-inducing conditions in the presence of 0.25 mM butyrate (*Ifnγ* and *Tbx21*) or 0.25 and 1 mM butyrate (*Foxp3*) was performed using an anti-acetyl-H3 antibody. For the ChIP analysis at the *Foxp3* promoter, naïve CD4$^+$ T cells were used as the control. *, P = 0.01-0.05, **, P = 0.001-0.01, n.s. = not significant. Two experiments were performed.

Supplementary Figure 6

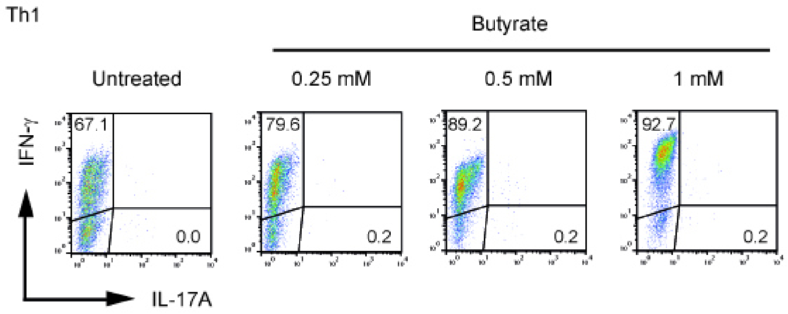

Supplementary Figure 6. Representative dot blots showing IFN-γ and IL-17A expression in $CD4^+$ T cells cultured under Th1 conditions for 6 days in the presence of indicated butyrate concentrations. Three independent experiments were performed.

Supplementary Figure 7

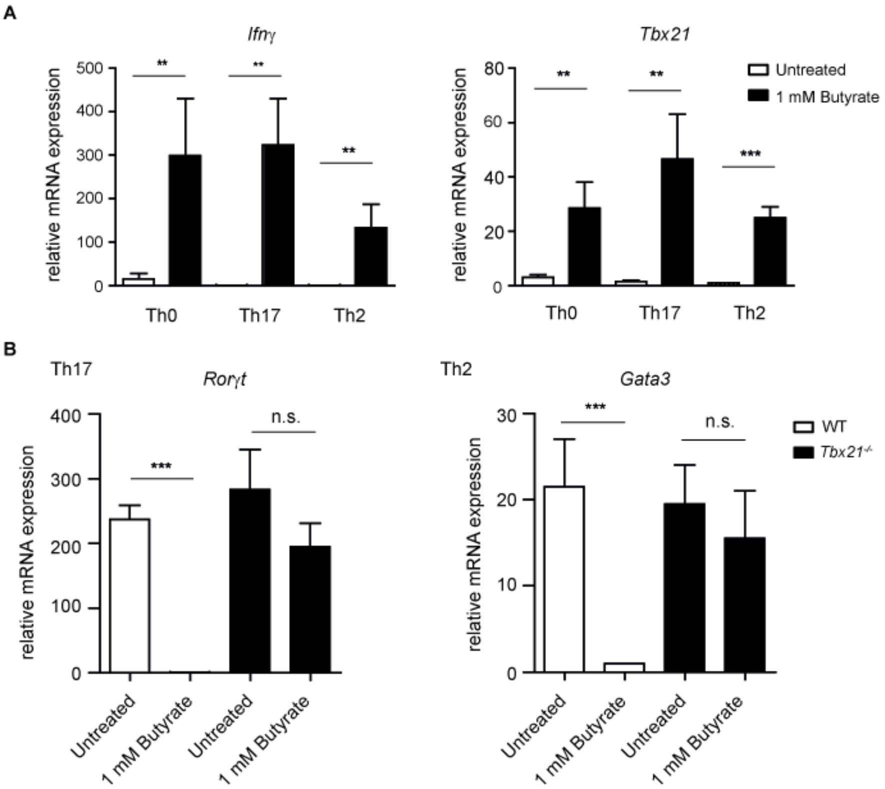

Supplementary Figure 7. (**A**) Impact of butyrate on the expression of *Ifnγ* and *Tbx21* in non-polarized and polarized WT $CD4^+$ T cells was anylased by RT-PCR. (**B**) WT and *Tbx21*$^{-/-}$ $CD4^+$ T cells were cultured under Th17- or Th2-polarizing conditions for 6 days in the absence or presence of butyrate. After 6 days of the cell cullture, the qRT-PCR analysis of *Rorγt* (Th17 cells) and *Gata3* (Th2 cells) was performed. Data are displayed as the mean ± SEM from two experiments; **, P = 0.001-0.01, ***, P <0.001., n.s. = not significant.

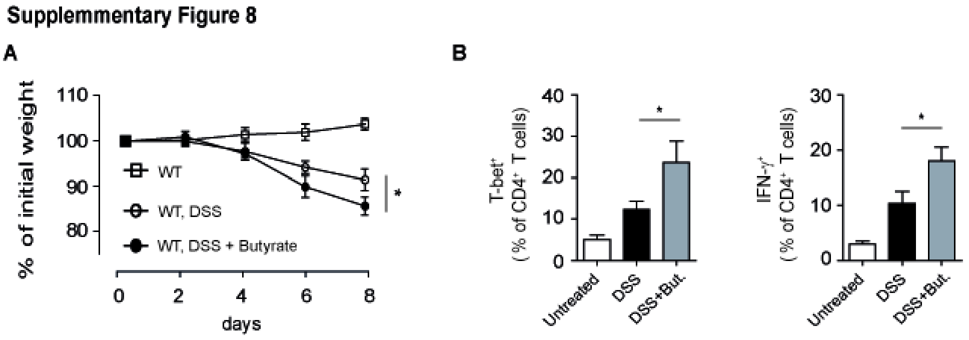

Supplementary Figure 8. (**A**) WT mice were orally given 2% DSS into the drinking water for 5 days. Weight loss was monitored each day throughout the course of the experiment; *, P = 0.01-0.05. Two experiments were performed. (**B**) The percentage of T-bet$^+$ (left) and IFN-γ^+ (right) cells within the colonic CD4$^+$ T cell population was analysed by flow cytometry. Two independent experiments were performed; *, P = 0.01-0.05

Supplemental Table 1

Colonic content	*GF mice*	*GF mice + DSS*	*GF +DSS + 100 mM Butyrate*
Acetate	-	-	-
Propionate	-	-	-
Butyrate	-	-	3,8 ± 0.48

Supplemental Table 1. Colonic contents (µmol/g luminal content) measured in the indicated experimental groups.
